# Sleep fragmentation and working memory in healthy adults

**DOI:** 10.5935/1984-0063.20200088

**Published:** 2021

**Authors:** Masato Okuda, Akiko Noda, Sho Mabuchi, Kunihiro Iwamoto, Masahiro Banno, Seiko Miyata, Fumihiko Yasuma, Norio Ozaki

**Affiliations:** 1 Chubu University Graduate School of Life and Health Sciences, Department of Biomedical Sciences - Kasugai - Aichi - Japan.; 2 Chubu University Collage of Life and Health Sciences, Department of Biomedical Sciences -Kasugai - Aichi- Japan.; 3 Nagoya University Graduate School of Medicine, Department of Psychiatry - Nagoya - Aichi -Japan.; 4 Seichiryo Hospital, Department of Psychiatry - Nagoya - Aichi - Japan.; 5 National Hospital Organization Suzuka Hospital, Department of Internal Medicine - Suzuka - Mie -Japan.

**Keywords:** Sleep Fragmentation, Sleep Stage, Wake After Sleep Onset, Working Memory, Cognitive Function

## Abstract

**Introduction:**

Sleep is essential for performing cognitive function in humans. We have hypothesized that sleep fragmentation compared to sleep efficiency may have a negative impact on the working memory.

**Material and Methods:**

Twenty-eight healthy adults (18 males and 10 females; mean age 27.8±15.5 years) were enrolled in this study. We measured the total sleep time (TST), sleep efficiency, %stage wakefulness (W), %stage rapid eye movement (REM), %stage N1, %stage N2, %stage N3, wake after sleep onset (WASO), and arousal index using polysomnography. Working memory, executive function, and sustained attention of three domains of cognitive function were evaluated with the number of back task (N-back task), Wisconsin card sorting test (WCST), and continuous performance test-identical pairs (CPT-IP), respectively.

**Results:**

The percentage of correct answers on the 2-back task was significantly correlated with %stage REM, %stage N1, and %stage N2 (%stage REM: *r*=0.505, *p*=0.006; %stage N1: *r*=-0.637, *p*<0.001; %stage N2: *r*=0.670, *p*<0.001), and multiple regression analysis including the stepwise forward selection method revealed that %stage N2 was the most significant factor (%stage N2: *β*=0.670, *p*<0.001). The percentage of correct answers on the 2-back task was also significantly correlated with TST, sleep efficiency, WASO, and arousal index (TST: *r*=0.492, *p*=0.008; sleep efficiency: *r*=0.622, *p*<0.001; WASO: *r*=-0.721, *p*<0.001; arousal index: *r*=-0.656, *p*<0.001), and WASO was the significant factor (*β*=-2.086, *p*=0.007). The WCST category achievement and CPT-IP *d*-prime score were correlated with none of the sleep variables.

**Conclusion:**

Increased WASO and a decrease in %stage N2 were associated with worse working memory.

## INTRODUCTION

Sleep plays an important role in mediating multiple domains of cognitive function in humans^1^. Sleep restriction is a facet of modern life that jeopardizes the cognitive performance including lapses of attention, slowed working memory, reduced cognitive throughput, and perseveration of thought^2^. An epidemiological study demonstrated that short and long sleep duration were associated with both objectively assessed and self-reported decreases in cognitive function in the general population^3^. In particular, the adverse effects of a short sleep duration on cognitive function have been well studied^2-5^. However, there has been no consensus regarding the impacts of sleep structure and sleep fragmentation on working memory.

The two main sleep states, i.e., the non-rapid eye movement (NREM) stages of N2 and N3 and rapid eye movement (REM) stage, have specific roles in sleep-dependent memory processing. Stage N3 and stage REM, as well as stage N2, are involved in memory consolidation^6^. Regarding the specific patterns of neural oscillation associated with each sleep stage, the ponto-geniculo-occipital waves and theta rhythms in stage REM, sleep spindles in stage N2, and slow wave activity of stage N3 have been postulated^7,8^. Moreover, an improvement in the visual discrimination task is correlated with the levels of stage REM^9^, while an improvement in the finger-tapping task is correlated with stage N2^10^. Although sleep fragmentation owing to frequent arousals during sleep likely contributes to the increased risk of cognitive decline, the impact of frequent arousals from sleep on cognitive function has not been systematically analyzed.

We previously showed that a short sleep duration had a negative impact on three domains of cognitive function, namely working memory, executive function, and sustained attention in young adults^11-13^ using the number of back task (N-back task)^14,15^, Wisconsin card sorting test (WCST)^16^, and continuous performance test-identical pairs (CPT-IP)^17^, respectively. The behavioral, cognitive, and psychophysiological effects of total/partial sleep deprivation or extended wakefulness have been well-documented^18^. There is a substantial variability of the results in sensitivity to sleep loss across the three cognitive domains. For example, the largest performance decrements are observed for measures of sustained attention and working memory^19^.

We have hypothesized that sleep fragmentation compared to sleep efficiency may have a negative impact on the working memory. Accordingly, we investigated the effects of sleep fragmentation and sleep stage on the three domains of cognitive function, i.e., the working memory, executive function, and sustained attention in healthy adults.

## MATERIAL AND METHODS

### Subjects

Twenty-eight healthy adults (18 males and 10 females; mean age 27.8±15.5 years) were enrolled in this study. None of the subjects had any history of neurological disorder, substance abuse, head injury, or major physical illness nor were they prescribed any psychotropic medications at the time of the study. This study (No. 270098) was approved by the ethics committee of Chubu University. Written informed consent was obtained from all participants after the nature of the study and procedures involved were fully explained.

### Polysomnography (PSG)

After one night’s acclimation, the subjects underwent the standard PSG with Alice 5 (Philips Respironics, Murrysville, PA) in accordance with the Manual of American Academy of Sleep Medicine (version 2.1)^20^. The PSG was conducted in the sleep laboratory at Chubu University. For each subject, lights out clock time was determined according to their habitual bedtime. Wake up time had to be adjusted to be the subjects’ needs until lights on clock time at 7:00 a.m. We obtained recordings of 6-channel electroencephalography (F_3_-M_2_, F_4_-M_1_, C_3_-M_2_, C_4_-M_1_, O_1_-M_2_, and O_2_-M_1_ electrodes), right and left electrooculogram, submental electromyograms, and electrocardiograms. We also used a microphone to record snoring and a sensor to monitor body position. Subsequently, we calculated the total sleep time (TST), sleep efficiency [TST/total recording time (TRT) × 100, %], %stage wakefulness (W) [W / sleep period time (SPT) × 100, %], %stage REM, %stage N1, %stage N2, and %stage N3.

The sleep stage in every 30 seconds of 1 epoch was scored according the criteria of the Manual of American Academy of Sleep Medicine (version 2.1)^20^. An epoch of stage N1 was scored if the majority of the epoch met the criteria for stage N1 (electroencephalogram showing low-amplitude, mixed-frequency electroencephalogram activity) in the absence of evidence for another sleep stage. An epoch of stage N2 was scored if the majority of the epoch met the criteria for stage N2 (K complex, sleep spindle). An epoch of stage N3 was scored when ≥20% of an epoch consists of slow wave activity. An epoch of stage REM was scored if the majority of the epoch met the criteria for stage REM (rapid eye movements, low chin electromyogram tone, sawtooth waves, transient muscle activity)^20^. Moreover, we evaluated the wake after sleep onset (WASO) and arousal index to quantify sleep fragmentation. Sleep onset was defined as the start of the first epoch scored as any stage in 30-second other than stage W. The WASO was defined as the sum of all waking hours during the sleep period and includes all wake activity, including time out of bed. Arousal was scored during stage N1, N2, N3 or REM if there was an abrupt shift of electroencephalogram frequency including alpha, theta and/or frequencies greater than 16 Hz (but not spindles) that lasted at least 3 seconds, with at least 10 seconds of stable sleep preceding the change. The arousal index was defined as the number of arousals per hour^20^.

### Cognitive function tests

In the morning after undergoing PSG, the subjects performed the N-back task^14,15^, WCST^16^, and CPT-IP^17^ to assess working memory, executive function, and sustained attention, respectively.

### N-back task

The participants underwent the N-back task to assess their working memory. In this task, they were required to update the mental set continually while responding to the stimuli (i.e., numbers) which were previously seen^14,21,22^. Each test comprised the 14 trials, each of which had a stimulus duration of 0.4s, an inter-stimulus interval of 1.4s, and 0-, 1-, and 2-back conditions. The stimuli consisted of numbers (2, 4, 6, or 8) shown in random sequence, which were displayed at the points of a diamond-shaped box^21^. Participants were required to respond to the stimuli using the numeric keypad of a computer. Performance was measured as the % correct (Hits + Correct Rejections/Total Stimuli × 100) and mean reaction time for correct hits on the keypad. In the N-back tasks for assessing working memory, the study participants were asked to indicate the timing when the presented number was the same as a series of number stimuli which were previously displayed.

### WCST

The WCST (WCST-Keio F-S version, Japanese Stroke Data Bank, Japan) was used for assessing their executive functions, including the abilities of reasoning abstract and shifting cognitive strategies in response to changing environmental contingencies^16,23^. We measured the category achievement of the number of categories among a maximum of eight categories, for which six consecutive correct responses were required^24^.

### CPT-IP

The CPT-IP (Biobehavioral Technologies, Inc., New York, NY, USA) was used for assessing the sustained attention or vigilance, as described previously^17^. A series of four-digit stimuli was presented for a period of 50ms, with an inter-stimulus interval of 950ms. Each session comprised 150 trials, among which 30 target trials were required to respond. Two sessions (first and second test) were conducted, and the results of the second test were adopted. Performances were assessed using the signal detection index *d*-prime, which was a measure of discriminability computed from “hits” and “false alarms”.

### Statistical analysis

All data were expressed as mean±standard deviation. We performed the Pearson’s correlation analyses followed by multiple regression analysis based on a stepwise forward selection method to determine the independent parameters that correlated with the 0-, 1-, and 2-back tasks, WCST, and CPT-IP in relation to %stage REM, %stage N1, %stage N2, and %stage N3 or TST, sleep efficiency, WASO, and arousal index. A probability value of 0.05 was considered statistically significant. All statistical analyses were performed using the SPSS version 25.0 (IBM Corporation, Armonk, New York, USA).

## RESULTS

The PSG findings and parameters by the N-back task, WCST, and CPT-IP were summarized in Table 1.

**Table 1.** PSG findings and parameters by the N-back task, WCST, and CPT-IP.

Parameters	
**PSG**	
TST (min)	383.2 ± 64.4
Sleep efficiency (%TRT)	89.6 ± 9.3
Stage W (%SPT)	6.0 ± 7.2
Stage REM (%SPT)	16.7 ± 6.6
Stage N1 (%SPT)	20.0 ± 14.2
Stage N2 (%SPT)	51.5 ± 14.9
Stage N3 (%SPT)	5.7 ± 5.4
WASO (min)	28.3 ± 35.8
Arousal index (/h)	14.2 ± 13.5
**N-back task**	
0-back task	
% corrects	99.4 ± 2.0
Reaction time (ms)	511.4 ± 99.5
1-back task	
% corrects	97.0 ± 4.6
Reaction time (ms)	426.0 ± 210.6
2-back task	
% corrects	87.0 ± 13.0
Reaction time (ms)	436.9 ± 224.1
**WCST**	
Category achievement	5.7 ± 0.8
**CPT-IP**	
Signal detection index (*d*-prime)	2.5 ± 0.9

Data are expressed as mean±standard deviation. PSG = Polysomnography; TST = Total sleep time; TRT = Total recording time; SPT = Sleep period time; WASO = Wake after sleep onset; WCST = Wisconsin card sorting test; CPT-IP = Continuous performance test-identical pairs.

### Correlation between the sleep parameters and N-back task

**Figure 1:**
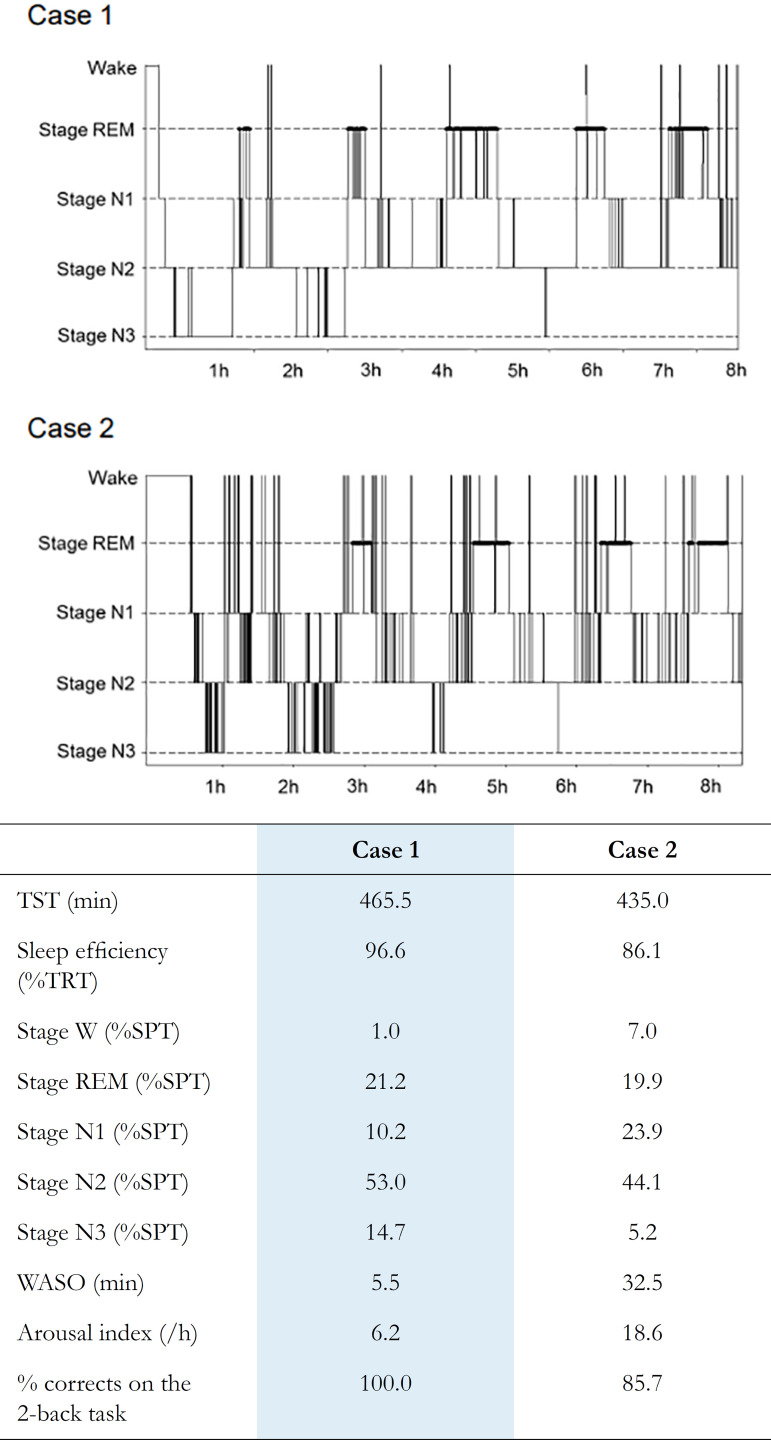
PSG hypnograms in representative case. WASO and arousal index were higher and the percentage of correct answers on the 2-back task was lower in case 2 compared to case 1. TST = Total sleep time; TRT = Total recording time; SPT = Sleep period time; WASO = Wake after sleep onset.

The percentage of correct answers on the 2-back task was significantly correlated with %stage REM, %stage N1, and %stage N2 (%stage REM: *r*=0.505, *p*=0.006; %stage N1: *r*=-0.637, *p*<0.001; %stage N2: *r*=0.670, *p*<0.001), and %stage REM and %stage N2 were the significant factors (%stage REM: *β*=0.559, *p*=0.025; %stage N2: *β*=1.010, *p*=0.039) (Table 2). Multiple regression analysis including the stepwise forward selection method revealed that %stage N2 was the most significant factor (%stage N2: *β*=0.670, *p*<0.001). The percentage of correct answers on the 2-back task was also significantly correlated with TST, sleep efficiency, WASO, and arousal index (TST: *r*=0.492, *p*=0.008; sleep efficiency: *r*=0.622, *p*<0.001; WASO: *r*=-0.721, *p*<0.001; arousal index: *r*=-0.656, *p*<0.001), and WASO was the significant factor (*β*=-2.086, *p*=0.007) (Table 3) (Figure 1). Multiple regression analysis based on a stepwise forward selection method including %stage REM, %stage N1, %stage N2, TST, sleep efficiency, WASO, and arousal index revealed that WASO was the most significant factor of the percentage of correct answers on the 2-back task (*β*=-0.721, *p*<0.001).

**Table 2.** Relationships among parameters by the N-back task, WCST, and CPT-IP and sleep stages.

	Simple correlation analysis		Multiple regression analysis		Simple correlation analysis		Multiple regression analysis	
	*r*	*p*	*β*	*p*	*r*	*p*	*β*	*p*
**0-back task**	% corrects				Reaction time			
Stage REM	0.282	0.145	0.148	0.645	-0.246	0.206	-0.394	0.196
Stage N1	-0.020	0.920	-0.603	0.476	0.405	0.032	-0.862	0.280
Stage N2	-0.231	0.237	-0.780	0.224	-0.516	0.005	-1.103	0.072
Stage N3	0.252	0.195	-0.033	0.922	-0.035	0.862	-0.148	0.638
**1-back task**	% corrects				Reaction time			
Stage REM	0.202	0.302	0.057	0.871	-0.425	0.024	-0.529	0.046
Stage N1	-0.288	0.137	-0.155	0.866	0.589	0.001	-1.003	0.142
Stage N2	0.244	0.212	0.090	0.896	-0.637	<0.001	-1.276	0.017
Stage N3	0.166	0.397	0.039	0.915	-0.214	0.273	-0.313	0.247
**2-back task**	% corrects				Reaction time			
Stage REM	0.505	0.006	0.559	0.025	-0.507	0.006	-0.690	0.015
Stage N1	-0.637	<0.001	0.616	0.327	0.511	0.005	-0.815	0.253
Stage N2	0.670	<0.001	1.010	0.039	-0.523	0.004	-0.990	0.070
Stage N3	0.161	0.412	0.071	0.774	-0.123	0.532	-0.083	0.767
**WCST**	Category achievement							
Stage REM	0.186	0.343	-0.294	0.359				
Stage N1	-0.290	0.134	-1.808	0.039				
Stage N2	0.220	0.260	-1.109	0.086				
Stage N3	-0.020	0.920	-0.706	0.043				
**CPT-IP**	*d*-prime							
Stage REM	-0.163	0.408	-0.021	0.951				
Stage N1	0.206	0.292	0.100	0.914				
Stage N2	-0.126	0.524	-0.011	0.988				
Stage N3	-0.218	0.266	-0.150	0.687				

WCST = Wisconsin card sorting test; CPT-IP = Continuous performance test-identical pairs.

**Table 3.** Relationships among parameters by the N-back, WCST, and CPT-IP and TST, sleep efficiency, WASO, and arousal index.

	Simple correlation analysis		Multiple regression analysis		Simple correlation analysis		Multiple regression analysis	
	*r*	*p*	*β*	*p*	*r*	*p*	*β*	*p*
**0-back task**	% corrects				Reaction time			
TST	0.217	0.268	0.558	0.050	-0.342	0.075	0.101	0.680
Sleep efficiency	-0.087	0.659	-0.133	0.831	-0.527	0.004	0.080	0.886
WASO	0.097	0.622	0.277	0.788	0.571	0.002	0.661	0.475
Arousal index	0.082	0.678	0.084	0.907	0.552	0.002	0.057	0.930
**1-back task**	% corrects				Reaction time			
TST	0.363	0.058	0.377	0.149	-0.568	0.002	-0.119	0.565
Sleep efficiency	0.336	0.080	0.761	0.200	-0.691	<0.001	-0.308	0.517
WASO	-0.238	0.222	-0.028	0.977	0.702	<0.001	-0.045	0.954
Arousal index	-0.157	0.424	0.803	0.242	0.700	<0.001	0.388	0.481
**2-back task**	% corrects				Reaction time			
TST	0.492	0.008	0.066	0.729	-0.434	0.021	-0.026	0.908
Sleep efficiency	0.622	<0.001	-0.757	0.093	-0.554	0.002	0.514	0.325
WASO	-0.721	<0.001	-2.086	0.007	0.637	<0.001	1.294	0.137
Arousal index	-0.656	<0.001	0.724	0.162	0.609	0.001	-0.197	0.742
**WCST**	Category achievement							
TST	0.147	0.455	0.301	0.288				
Sleep efficiency	-0.009	0.963	-0.898	0.169				
WASO	-0.059	0.767	-1.524	0.157				
Arousal index	-0.019	0.926	0.859	0.253				
**CPT-IP**	*d*-prime							
TST	-0.319	0.098	-0.449	0.117				
Sleep efficiency	-0.122	0.536	0.482	0.451				
WASO	0.159	0.420	0.668	0.525				
Arousal index	0.146	0.458	-0.380	0.607				

TST = Total sleep time; WASO = Wake after sleep onset; WCST = Wisconsin card sorting test; CPT-IP = Continuous performance test-identical pairs.

The reaction time on the 1-back task was significantly correlated with %stage REM, %stage N1, and %stage N2 (%stage REM: *r*=-0.425, *p*=0.024; %stage N1: *r*=0.589, *p*=0.001; %stage N2: *r*=-0.637, *p*<0.001), and %stage REM and %stage N2 were the significant factors (%stage REM: *β*=-0.529, *p*=0.046; %stage N2: *β*=-1.276, *p*=0.017). The reaction time on the 2-back task was significantly correlated with %stage REM, %stage N1, and %stage N2 (%stage REM: *r*=-0.507, *p*=0.006; %stage N1: *r*=0.511, *p*=0.005; %stage N2: *r*=-0.523, *p*=0.004), and %stage REM was the significant factor (*β*=-0.690, *p*=0.015) (Table 2).

### Correlation between the sleep parameters and WCST or CPT-IP

The WCST category achievement and CPT-IP *d*-prime score were not correlated with %stage REM, %stage N1, %stage N2, %stage N3, TST, sleep efficiency, WASO, and arousal index.

## DISCUSSION

We found that increased WASO and a decrease in %stage N2 were associated with the lower percentages of correct answers on the 2-back task, and that WASO was the most significant factor influencing the percentages of correct answers on the 2-back task among three domains (working memory, executive function, and sustained attention) of cognitive function. Our findings suggest that increased WASO and a decrease in %stage N2 have a negative effect on the working memory.

Sleep fragmentation plays a vital role in the progression of cognitive decline by promoting endothelial dysfunction^25^ and inflammation^26^. Chronic sleep fragmentation model confirmed that preserved sleep quality in addition to sleep duration is essential for cognitive performance^27-29^. There are substantial changes with aging in the overall sleep quality, slow wave sleep, spindle density, and sleep fragmentation^30-32^. Sleep disturbance is a common symptom among young adults and is associated with such factors as decreased quality of life, chronic somatic diseases, and mental illnesses^33^. A previous study using actigraphy among older community-dwelling people showed that a longer TST and greater WASO were associated with lower cognitive performance^34^. Moreover, sleep fragmentation concomitant with surges in blood pressure and heart rate has been shown to result in daytime sleepiness that impairs psychomotor function even in healthy individuals^35^. Thus, increased WASO and frequent arousals lead to sleep fragmentation and poor sleep quality, which are likely to precipitate the cognitive decline in the long run.

In the present study, the percentage of correct answers on the 2-back task was significantly correlated with %stage N2. The reaction times on 0- and 1-back tasks were closely correlated with the %stage N2, which reflects visual-speed processing in synchrony with commands of motor output from the premotor area^36^. Stage N2 spindle density was reported to be a marker of neural connectivity of the brain, which was essential for maintaining the general cognitive function^37^. Schabus et al. (2006)^38^ divided sleep spindles into two categories: fast and slow. Slow spindles (<13Hz) predominate over the frontal cortical areas, whereas fast spindles (>13Hz) prevail over the parietal and central areas^39^. The spindles are associated with activity in the hippocampus^38^. They are temporally coupled with oscillations in the sharp wave ripple of the hippocampus during sleep^40^. Recently, an animal study demonstrated that working memory was associated with hippocampal neuronal activity^41^. Therefore, we believe that stage N2 may provide us with critical information for working memory. Future studies should address potential mechanisms underlying the relationship between %stage N2 and working memory.

We showed that the percentage of correct answers on the 2-back task significantly correlated with %stage REM. A recent PSG study in 58 healthy middle-aged and older adults demonstrated that REM sleep duration was associated with better learning potential of verbal memory, which suggest that longer REM sleep duration may be a marker of acetylcholine system integrity^42^. Moreover, the ability to maintain working memory was associated with REM duration and TST in the daytime nap in 80 healthy college students^43^. The effect of sleep loss on the working memory was found to relate to the degraded neural activation of the prefrontal cortex (PFC) and functional connectivity with other brain regions^44^. Although the mechanisms of the dynamic changes of the PFC activities from NREM to REM have remained unclear, REM sleep appears to play an important role in the selective activation and deactivation of PFC during sleep. Hence, sufficient REM sleep could preserve the enhanced working memory.

In this study, the percentage of correct answers on the 2-back task was not significantly correlated with %stage N3. Working memory provides a temporary storage space and the information necessary for processing tasks and acts as the bridge between the instantaneous and long-term memory systems in the human brain^45^. Using electroencephalography, a recent study in healthy male adults demonstrated that higher slow wave activity in the frontoparietal regions predicted a better working memory^46^. A PSG study in healthy middle-aged and older participants reported that slow wave density and slow wave slope were correlated with verbal fluency performance, but no significant correlations were found between 2-back performance and any sleep variables^42^. An intervention study on three weeks of working memory training in male children and adolescents showed that the increase in slow wave activity was correlated with cognitive training-induced plasticity in a region known to be involved in working memory performance^47^. The methodology of evaluation and ages of the subjects may influence the different effects of stage N3 on working memory.

The category achievement by the WCST was not correlated with %stage REM, %stage N2, and WASO. The category achievement was 6 in 57.1% of all participants, which may have created a ceiling effect due to the ease of completing the WCST in healthy adults. Regarding the difference between the WCST and N-back task of the frontal tasks, the N-back task is a test for working memory processes^14,15,48^, and the WCST is a test for executive function^16^. Hence, the differential role of the N-back task and WCST could explain the differences in our results for the two tasks. Moreover, the CPT-IP *d*-prime score, which reflects sustained attention, was not correlated with %stage REM, %stage N2, and WASO in this study. A previous PSG study showed that there were no significant correlations between the parameter by CPT-IP and sleep variables in healthy middle-aged and older participants^42^, which seems consistent with our findings.

Our study had a few limitations. First, our study population was relatively small. Second, we could not investigate the gender differences in the effect of sleep fragmentation on cognitive function owing to our small sample size. The sexes did not differ significantly on the N-back task in 93 healthy young subjects aged 17 to 32 years^49^. However, insufficient sleep may enhance anxiety in women. Women are more susceptible to the emotional consequences of sleep deprivation and sleep disruption than men^50,51^. Third, the current study is a cross-sectional study and it is not possible to determine whether WASO is the cause of cognitive decline. To assess the effects of sleep fragmentation on working memory, we could not evaluate the effects of arousals on working memory when subjects were randomly divided into two groups, with one group using, for example, auditory stimulation to induce sleep fragmentation and the other group allowing subjects to sleep uninterruptedly. Moreover, a clearer index of fragmentation is the number of arousals, even if TST is not changed. Another one could be the probability of transition from NREM to stage W^52^. Intervention trials with larger sample sizes may be required to elucidate the effect of sleep stage and sleep fragmentation on the working memory, executive function, and sustained attention in healthy adults of both genders.

## CONCLUSION

Among the three domains of cognitive function, increased sleep fragmentation and a decrease in %stage N2 were associated with worse working memory. Evaluation of the major domains of cognitive function along with PSG analysis could yield practical and theoretical implication for cognitive impairment.
